# Supplementary material for: Fast generation of Schrödinger cat states using a Kerr-tunable superconducting resonator
Source: Nat Commun. 2023 Oct 11;14:6358. doi: 10.1038/s41467-023-42057-0 (PMC10567735; doi:10.1038/s41467-023-42057-0)
Supplement: Supplementary file 1 — Supplementary Information [file 41467_2023_42057_MOESM1_ESM.pdf]

Supplementary Information - Fast generation of Schrödinger cat states using a Kerr-tunable superconducting resonator

X.L. He[1,2†], Yong Lu[3,4*†], D.Q. Bao[1,2], Hang Xue[1,2], W.B. Jiang[1,2], Z. Wang[1,2], A.F. Roudsari[4], Per Delsing[4], J.S. Tsai[5,6] and Z.R. Lin[1,2*]

[1]National Key Laboratory of Materials for Integrated Circuits, Shanghai Institute of Microsystem and Information Technology, Chinese Academy of Sciences, Shanghai, 200050, China.
[2]University of Chinese Academy of Science, Beijing, 100049, China.
[3]3rd Physikalisches Institut, University of Stuttgart, 70569, Stuttgart, Germany.
[4]Microtechnology and Nanoscience, Chalmers University of Technology, SE-412 96, Göteborg, Sweden.
[5]Graduate School of Science, Tokyo University of Science, Shinjuku, Tokyo, 162-0825, Japan.
[6]Center for Quantum Computing, RIKEN, Wako, Saitama, 351-0198, Japan.

*Corresponding author(s). E-mail(s): kdluyong@outlook.com; zrlin@mail.sim.ac.cn;
†These authors contributed equally to this work.

Supplementary Note 1 – Odd/Even Cat states generation

The cat states with odd/even properties are generated by following the pulse sequence in Supplementary Fig. 1(a) [1][2]. We firstly excite the qubit to $(|e\rangle + |g\rangle)/2$ by a $\pi/2$-pulse. Then, a displacement pulse is injected to initialize the SNAIL-terminated resonator in a coherent state. After a certain period of evolution τ ($\tau = \pi/\chi,\ 3\pi/\chi$). The entangled states can be written as:

$$|\Psi\rangle = \frac{1}{\sqrt{2}}|e\rangle \otimes |-\alpha\rangle - \frac{1}{\sqrt{2}}|g\rangle \otimes |\alpha\rangle,\ (\tau = \pi/\chi)$$
$$|\Psi\rangle = \frac{1}{\sqrt{2}}|e\rangle \otimes |-\alpha\rangle + \frac{1}{\sqrt{2}}|g\rangle \otimes |\alpha\rangle,\ (\tau = 3\pi/\chi) \tag{1}$$

After an additional displacement pulse $D(\alpha)$, the state becomes:

$$|\Psi\rangle = \frac{1}{\sqrt{2}}|e\rangle \otimes |0\rangle \pm \frac{1}{\sqrt{2}}|g\rangle \otimes |2\alpha\rangle. \tag{2}$$

Next, we apply a conditional π-pulse, which only works when the photon number is small ($< 4\alpha^2$).

$$|\Psi\rangle = |g\rangle \otimes (|0\rangle \pm |2\alpha\rangle). \tag{3}$$

Substantially, the two component cat states $|\alpha\rangle \pm |-\alpha\rangle$ are generated after the third displacement $D(-\alpha)$.

$$|\Psi\rangle = |g\rangle \otimes (|\alpha\rangle \pm |-\alpha\rangle). \tag{4}$$

The corresponding Wigner function in Supplementary Fig. 1(b) clearly show the interference among coherent states. The asymmetric pattern, highlighted by the dashed blue line in Supplementary Fig. 1(b), comes from the higher decoherence

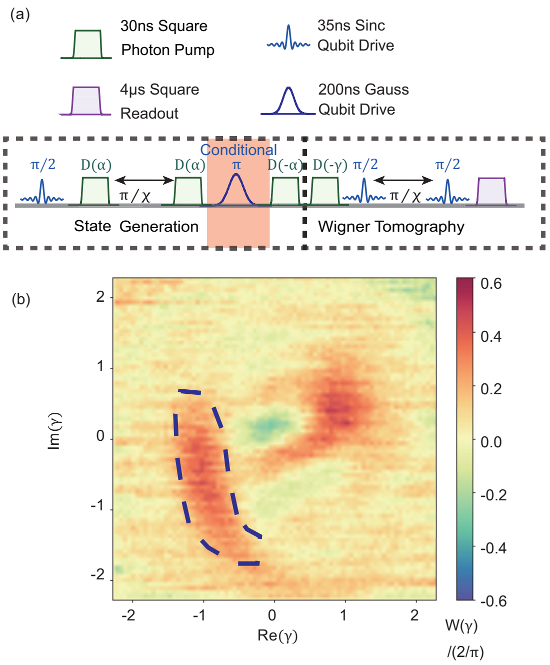

Supplementary Fig. 1. Odd/even cat generation. (a) Pulse sequence for odd/even cat preparation. (b) Measured Wigner function of an odd cat state $|\varphi_{-Z}\rangle = |\alpha\rangle - |-\alpha\rangle$, where α=1.42.

and dissipation rates under a larger photon number (Supplementary Fig. 2). In details, during a period of the state preparation (shadowed by red in Supplementary Fig. 1(a)), a part of the state is displaced to the position $|2\alpha\rangle$, with the photon number four time larger than the initial state $|\alpha\rangle$, leading to an enhanced decoherence and dissipation [1]. As a proof, the photon number dependent decoherence rate is measured (discussed in the next section).

Supplementary Note 2 – Photon decoherence and relaxation

As theoretical analysis [1], the decoherence and relaxation rate are highly affected by the photon number. The decoherent time of the light field (coherent states) is characterized by scanning the time spacing between two opposite displacement ($D(\alpha)$ and $D(-\alpha)$). In our system, we can clearly see the decoherent time T_2 drops while increasing the pump power (Supplementary Fig. 2). It

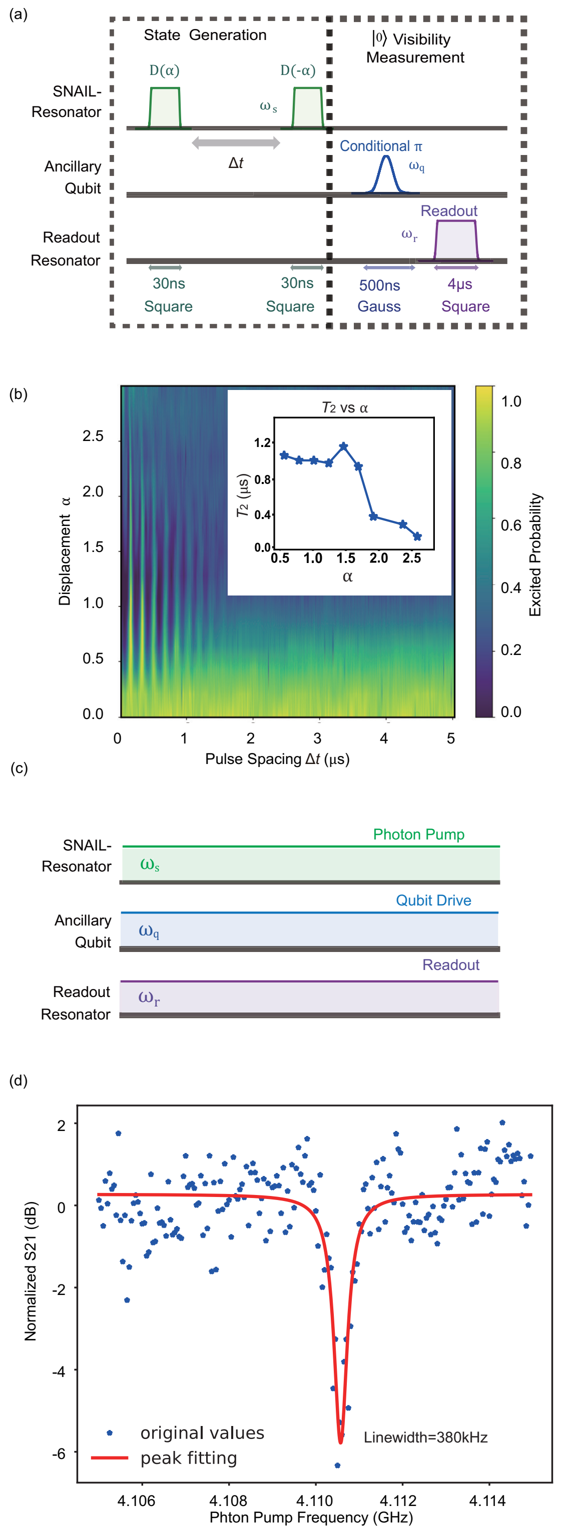

Supplementary Fig. 2. Relaxation and decoherence measurement of the SNAIL-terminated resonator. (a) Decoherence measurement pulse sequence. (b) The displacement (photon number) related T_2 measurement. (c) The continuous waves for linewidth test. (d) Spectroscopy measurement for characterizing the linewidth of the SNAIL-terminated resonator

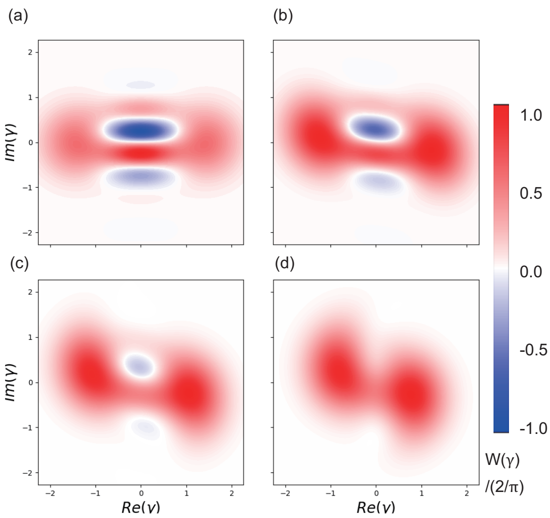

Supplementary Fig. 3. Simulated Wigner functions of a 2-component cat state with single photon loss and residual Kerr coefficient after (a) 0 ns. (b) 100 ns. (c) 200 ns. (d) 400 ns evolution

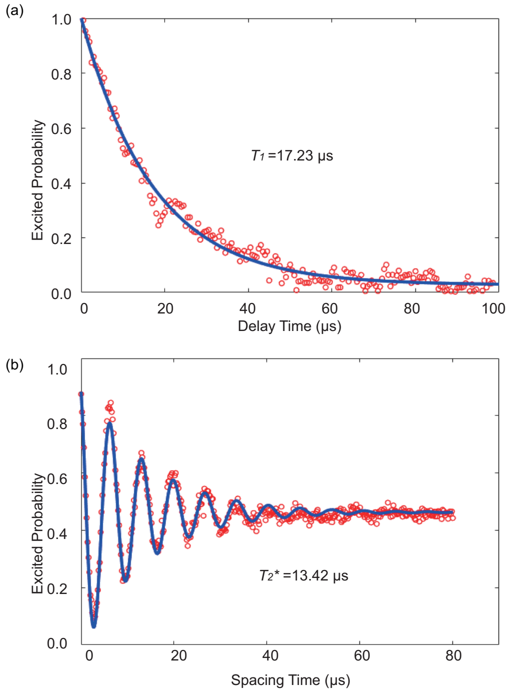

Supplementary Fig. 4. (a)T_1 and (b)$T_2{}^*$ of the ancillary qubit

is also the reason for the asymmetric shape in Supplementary Fig. 1(b).

The linewidth of the SNAIL terminated resonator is measured with continuous waves (Supplementary Fig. 2(c)). By injecting photons through the charging line with low power (-140 dBm), the linewidth of the nonlinear resonator is 380 kHz(Supplementary Fig. 2(d)). In Supplementary Fig. 3, we simulate how a 2-component cat state evolves under a lossy and nonlinear Hamiltonian similar with our case (with $\kappa/2\pi = 200$ kHz single photon loss rate and $K/2\pi = 50$ kHz Kerr coefficient). Since the single loss may completely damage the parity property of the cat state, the interference fringes disappear within 400 ns. To improve the performance of the system, the quality factor (around 10^5 so far) can be improved by optimized designs and fabrication processes.

Supplementary Note 3 – Relaxation and decoherence of the ancillary qubit

The relaxation and decoherence features of the ancillary qubit are shown in Supplementary Fig. 4. Considering the coupled nonlinear resonator, a 10 μs-long lifetime is resonable and acceptable to demonstrate the operations discussed above.

Supplementary Note 4 – Measurement setup

The sample is mounted inside a dilution refrigerator (BlueFors LD400) system, the schematic diagram is shown in Supplementary Fig. 5. We use 3 layers of μ-metal to shield the magnetic noise. A superconducting coil installed upon the sample holder to produce the static magnetic field. To amplify the output signal, we use three-level amplification– a Josephson parametric amplifier (JPA) with Nb trilayer Josephson junctions [3] at 10 mK, a high electron mobility transistor (HEMT) at 4 K and a low-noise amplifier at room temperature.

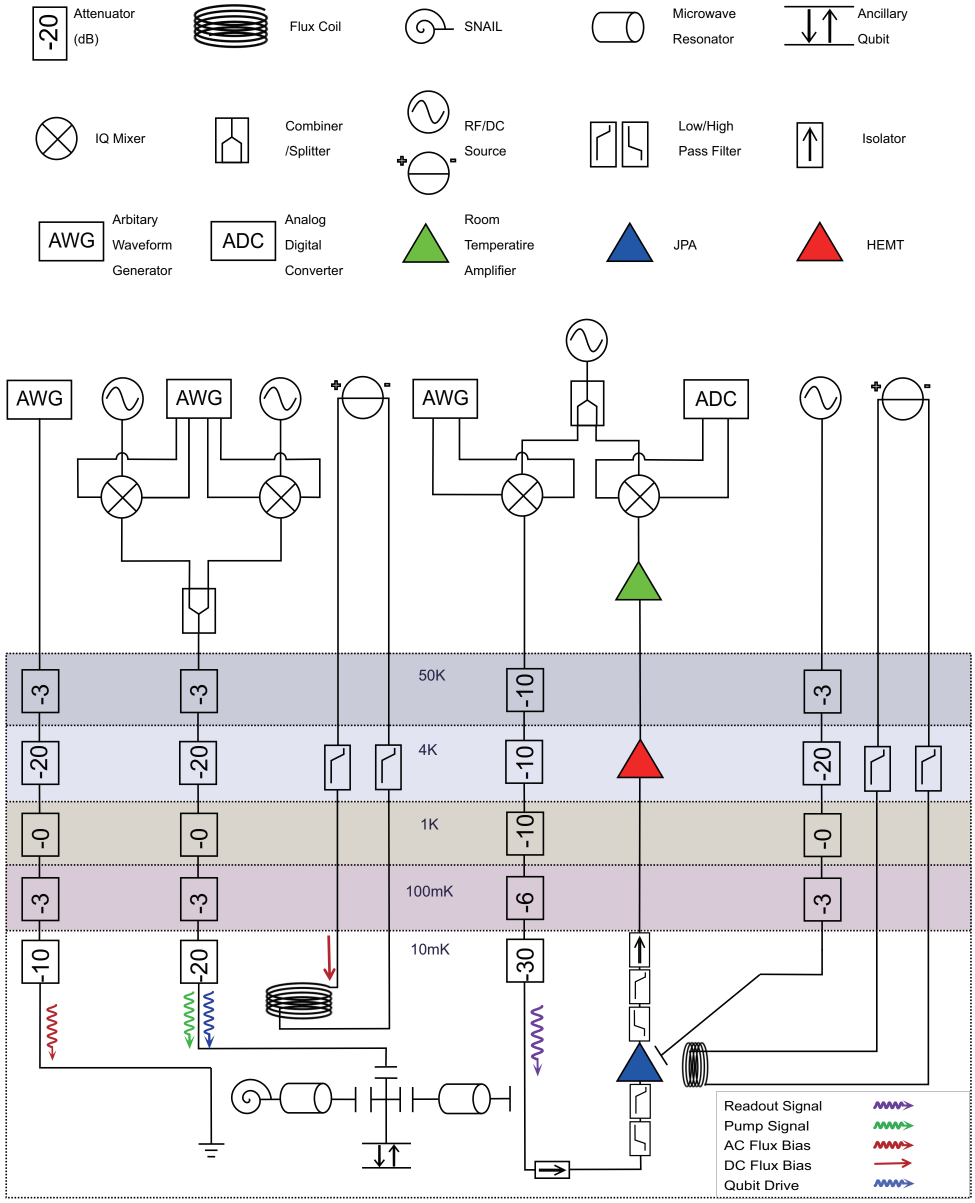

Supplementary Fig. 5. Schematic diagram of the measurement system.

References

[1] Serge Haroche and J. Raimond. *Exploring the Quantum: Atoms, Cavities, and Photons.* Oxford Univ Pr, 2006.

[2] Chen Wang, Yvonne Gao, Philip Reinhold, et al. A Schrodinger Cat Living in Two Boxes. *Science*, 352, 01 2016.

[3] Hang Xue, Zhirong Lin, Wenbing Jiang, et al. Fabrication and characterization of all-Nb lumped-element josephson parametric amplifiers. *Chinese Physics B*, 30, 03 2021.
